# An Intra-Vehicular Wireless Multimedia Sensor Network for Smartphone-Based Low-Cost Advanced Driver-Assistance Systems

**DOI:** 10.3390/s22083026

**Published:** 2022-04-15

**Authors:** Christiaan M. Fourie, Hermanus Carel Myburgh

**Affiliations:** Department of Electrical, Electronic and Computer Engineering, University of Pretoria, Pretoria 0002, South Africa; herman.myburgh@up.ac.za

**Keywords:** ADAS, ADAS and smartphones, IVWSN, object detection, WMSN

## Abstract

Advanced driver-assistance system(s) (ADAS) are more prevalent in high-end vehicles than in low-end vehicles. Wired solutions of vision sensors in ADAS already exist, but are costly and do not cater for low-end vehicles. General ADAS use wired harnessing for communication; this approach eliminates the need for cable harnessing and, therefore, the practicality of a novel wireless ADAS solution was tested. A low-cost alternative is proposed that extends a smartphone’s sensor perception, using a camera-based wireless sensor network. This paper presents the design of a low-cost ADAS alternative that uses an intra-vehicle wireless sensor network structured by a Wi-Fi Direct topology, using a smartphone as the processing platform. The proposed system makes ADAS features accessible to cheaper vehicles and investigates the possibility of using a wireless network to communicate ADAS information in a intra-vehicle environment. Other ADAS smartphone approaches make use of a smartphone’s onboard sensors; however, this paper shows the application of essential ADAS features developed on the smartphone’s ADAS application, carrying out both lane detection and collision detection on a vehicle by using wireless sensor data. A smartphone’s processing power was harnessed and used as a generic object detector through a convolution neural network, using the sensory network’s video streams. The network’s performance was analysed to ensure that the network could carry out detection in real-time. A low-cost CMOS camera sensor network with a smartphone found an application, using Wi-Fi Direct, to create an intra-vehicle wireless network as a low-cost advanced driver-assistance system.

## 1. Introduction

Vision from inside vehicles is becoming more common, especially in autonomous vehicles. Sensory networks used within vehicles, and how their applications improve the awareness of vehicles on the road, were investigated. Smartphones are used in forward, lateral, and inside assistance ADAS applications, in object and lane detection, tracking, and traffic sign detection. Forward assistance includes autonomous cruise control (ACC), which assists drivers in automatically keeping a safe driving distance, and forward collision avoidance (FCA), which provides a warning to the driver in the event of a potential accident. Forward assistant methods use radar and LiDAR. FCA also uses sensors, such as radar and LiDAR, but car manufacturers are using video in conjunction with radar, which has opened up a wide range of topics in research studies, i.e., using image processing in object detection, while sensor fusion techniques can be used to complement sensor devices for vehicle detection [1,2,3,4].

Smartphones are cheaper alternatives to forward assistance ADAS. Many methods of monitoring road and traffic conditions, using smartphones, have been proposed; the first approaches used sensors, three-axis accelerometers, and GPS [5,6,7,8]. Vehicle detection has been conducted using image processing, as well as alternatives to radar and LiDAR by using local binary patterns (LBP) and Haar-like features to train AdaBoost classifiers [9]. Monocular vision avenues have been investigated, detecting pedestrians and vehicles, using Haar-like features and “cascaded” weak classifiers with distance estimation [10]. In [11], stereo vision involved two cameras carrying out 3D reconstruction, in such a way that the cameras were calibrated to compute extrinsic parameters to calculate the necessary coordinate system in front of the vehicle.

A technique used extensively by ADAS in smartphones is the Viola–Jones algorithm, which uses a sliding window over the whole image, looks for Haar-like features selected by AdaBoost, and uses weak classifiers to detect objects [12]. Object detection using convolutional neural networks (CNNs) is used in smartphones that use pre-trained CNNs [13]. Object detection using CNNs is improving, since the R-CNN inception—which uses a region proposal to extract potential bounding-boxes to run classifiers on these boxes, instead of scanning the whole image to you only look once (YOLO)—uses the entire image. It divides the image, predicts bounding-boxes and their confidence in being objects, and then predicts class probabilities. The images are analysed, and predictions are informed by the global context of the image [14,15,16,17]. YOLO was shown to be the fastest real-time detector on PASCAL VOC at speeds of up to 155 FPS for fast YOLO, which is currently the best general detector that detects a variety of objects simultaneously and performs well to detect vehicles and other objects, with its superior precision to recall ratio [17]. The mentioned CNN-trained models are too large for portable mobile devices and are improved by using MobileNets that use depth-wise separable convolutions to build lightweight deep neural networks [18].

Lane detection ensures that the vehicle orientates between lanes and, if required, keeps the vehicle within the middle of the lanes by warning the driver if the vehicle is diverging. Different approaches to lane detection by using smartphones exist, such as LaneQuest, which uses the smartphone’s GPS and inertial sensors [19]. DeepLane uses GPS and camera-assisted vision from the camera at the back of the smartphone to estimate the vehicle’s lane position [13]. Lane detection technologies, such as SmartLDWS, showed successful lane tracking, consisting of a lane detection module that used image processing to conduct edge detection through the Hough transformation, where the processing was conducted on a smartphone attached to a vehicle’s windscreen, thereby facing the front of the vehicle [20]. The real-time lane detection was also conducted in [21], with the addition of a rear-end collision warning by using the camera of the smartphone to detect vehicles and their distances, using the Canny edge detector and triangulation, respectively. Other vehicle detection techniques implemented on the DriveSafe application, initially developed for scoring driver behaviours [22], were expanded by using different detection windows to detect vehicles with the help of AdaBoost classifiers and tracking, using Kalman filters [9]. DeepLane ignores road lanes entirely, which is beneficial in rural areas or crowded traffic lanes where lines are not visible. DeepLane does not track the lanes but uses optical flow (OF) to detect the flow of pixels and the flow of objects where the objects are detected, using YOLO [17].

Lateral assistance in ADAS attempts to avoid collisions with vehicles in the driver’s blind spot or when the driver is unable to anticipate the speed of (and the possible collision with) another vehicle outside of the driver’s view. IVWSNs have been used to develop low-cost substitutes of side blind zone alert (SBZA) systems that monitor blind spots of vehicles and alert users. An IVWSN uses Bluetooth Low Energy (BLE), which is a low-cost and low-power Bluetooth technology [23]. Blind spot detection, using cameras pointing in the area of a vehicle’s blind spots, has been studied, showing valid detection results using image processing techniques from the cameras mounted on side mirrors of vehicles [24,25,26]. The application of smartphone involvement with blind-spot detection is limited: SideEye attempted blind-spot detection on the driver’s side of the car, using the front camera of the smartphone. The front camera captures some of the blind-spot areas where image processing is used to detect other vehicles [27].

Inside assistance includes analysing driving behaviour, such as drowsiness, sleepiness, and fatigue that could be detrimental to the driver [28]. A seminal paper on driver monitoring for intelligent vehicles categorises causes of inattention as a distraction and fatigue [29]. Smartphone-embedded hardware and cost-efficiency benefits have been used to address these driving behaviours [10,30,31,32,33]. Algorithms are used to detect driving events while using the front camera of the smartphone to monitor the driver’s facial expressions and gestures; intervention can be triggered before an accident occurs [34]. CarSafe was the first driver safety smartphone application on smartphones to use dual cameras where the front camera was used for monitoring the driver and the rear camera to detect the following distance and lane drifting [10].

ADAS applications on smartphones have been implemented, using iOS, as well as Android operating systems, including CarSafe, SideEye, and DeepLane [9,10,13,27]. Android smartphones have an extensive range of hardware combinations, but as shown by DeepLane, smartphones with better GPUs run at higher frame rates, making real-time applications reachable [13].

Wired architectures in vehicles are the most traditional, but new wireless network alternatives exist, such as IVWSN, which are being considered by the automotive industry. With the help of wireless technologies, the vehicle’s harnessing is reduced, thereby lowering costs and fuel consumption [23]. CAN are shown to have bandwidth limitations on the CAN bus with the introduction of camera-based ADAS [35]. Other wired networks used in vehicles exist with varying bitrates, which have higher bandwidths and are capable of transmitting vision information, such as media-oriented system transport (MOST) [36]. IEEE 802 and ultra-wideband (UWB) solutions are currently being investigated, but because they still require electrical power sources from the vehicles, the advantages are mitigated [35]. However, the inner vehicle is a challenging environment for radio propagation because of metal objects and passengers inside the vehicle [37]. As a result of this complex environment, design considerations should be carefully considered when developing a wireless sensor network inside a vehicle where the reliability of the network diminishes as traffic on the network increases, causing delays in information being transferred [38,39]. Currently, different network compatible devices are available, e.g., Wi-Fi, Bluetooth, UWB, and Zigbee.

An IoT-IVWSN network consisting of end-devices, control unit, and a display that has a large number of end-device sensors, was shown to be a good alternative in vehicles to control and manage sensors [39]. Zigbee technology was shown to be useful for sensors that do not require high data transmission rates. However, when transferring multimedia and real-time video streaming, it would not be a practical solution. UWB and Wi-Fi are better solutions for high data rate implementations, such as multimedia video, because of the high data rate and low power [40]. A network of wirelessly interconnected devices that retrieve video and audio streams is known as wireless multimedia sensor networks (WMSN), where low-cost CMOS cameras, supplying multiple media streams, can provide a multi-resolution description of scenes. Sensors that are capable of collecting multimedia data require computation-intensive processing, which may require processing to be conducted on the sensor nodes or computational hubs where WMSNs enable a new approach to perform distributed computations on nodes [41].

Vision-based sensors have become very popular in ADAS because of their low costs, compared to radar and LiDAR, but vision-based sensors perform poorly in unfavourable weather conditions. Measuring the distance of an object using vision sensors is also less accurate than LiDAR and radar, whereby using stereo vision (dual cameras) requires a wide base length between cameras for triangulation purposes; a radar’s robustness against weather and the ability to determine distance more accurately can be harnessed instead, and is shown to work well in multi-sensor scenarios that lower false positives in detection [2,42,43].

ADAS depend on sensor information from the surrounding environment, processed by the electronic control unit (ECU) in high-end vehicles. These sensors include radar, LiDAR, ultrasonic sensors, and IR sensors, and the accommodate ADAS features, such as lane departure warning, parking assistance, and blind spot monitoring [44,45]. Ultrasonic sensors are among the cheapest sensors in ADAS, which are primarily used to find distances of static objects at a short-range and at slower speeds [44]. Ultrasonic sensors have a shorter range than radar, limited to a range of 2 metres, but could serve as a cheaper, less power consuming alternative for low-cost ADAS.

**Figure 1:**
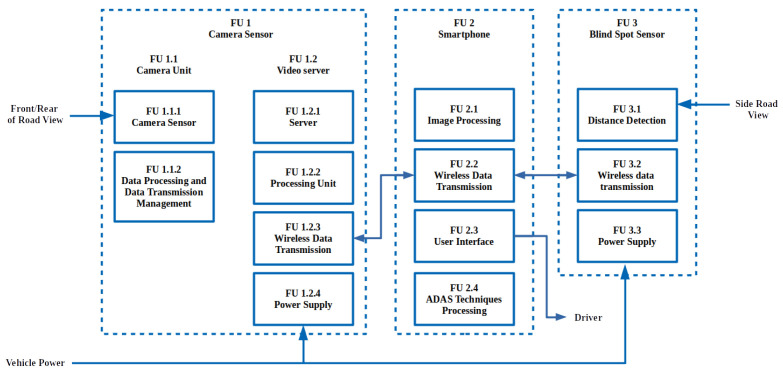
Functional diagram of a proposed wireless ADAS, using a smartphone.

In this paper, we propose using a wireless ADAS, as shown in Figure 1; the ECU and display unit in a high-end ADAS are merged into FU 2, consisting of a smartphone. The sensors, FU 1 and FU 3, communicate with the smartphone through a wireless network where the ADAS techniques and processing are carried out (FU 2.4). By using the standard connection capabilities, the network can consist of Wi-Fi and Bluetooth protocols. Wi-Fi is a better-suited protocol for transferring information from camera sensors where an IP network is required to establish communication. The smartphone in FU 2.2 becomes an access point (AP), and the camera sensor’s hosted video server allows the smartphone to access video through the Wi-Fi network (FU 1.2).

The network also consists of blind spot sensors that are not as data-intensive as the camera sensors. The blind spot sensors only require the distances to be sent to the smartphone and require minimum data throughput. FU 3.2 is used by the blind spot sensor to transfer data to the smartphone. Finally, the display and audio of the smartphone are used to interact with the driver, warning about potential risks.

There is no active research on WMSN for ADAS applications, and its collaboration with smartphones is non-existent. Complementary metal-oxide semiconductor (CMOS) camera sensors have become more affordable and were shown to be helpful in ADAS, but they only exist as wired solutions; a wireless network of vision sensors has research potential [46]. Ultra-wideband (UWB) and Wi-Fi have high transmission rates that can be used for video transfer in smartphones, making them excellent additions to vision sensor networks. UWB would be the ideal choice because of the low power usage, high transmission rate, and low cost of UWB. However, UWB modules in smartphones have only recently become commercially available in high-end models, such as the iPhone 11, subsequently leading to new research avenues. An intra-vehicular wireless sensor network (IVWSN) can expand a smartphone’s limited sensor perception to a cost-efficient, camera-based ADAS that uses real-time object detection to detect potential hazards around the vehicle’s rear, blind spots, and front spatial areas.

The ADAS is wholly dependent on the data received by the IVWSN. Wireless technologies available on smartphones are considered, namely Wi-Fi and Bluetooth. This paper is organised as follows, in Section 2, these two wireless technologies are explored to use their capabilities and technology stacks that sensors will then implement in the IVWSN. Network topologies for multiple sensor uses are examined, followed by detailed communication methods and protocols to communicate with different sensors in the network. Detailed designs of the proposed camera nodes that use video streaming through the network are presented. Additional sensory data are also provided via a detailed design of the blind spot nodes. A comprehensive explanation of a developed smartphone ADAS application follows the hardware development, realising the complete solution. Different ADAS techniques implemented on the smartphone are shown with detailed explanations.

In the concluding sections, the systems being designed and developed, are discussed. The network’s results are divided into: simulated environment, inta-vehicle environment, and power consumption. Simulated and actual environments are shown, which are compared with controlled environments and actual intra-vehicle counterparts. The performance results of the ADAS techniques implemented on the smartphone are illustrated and discussed for lane, collision, and blind spot detection.

## 2. Materials and Methods

**Figure 2:**
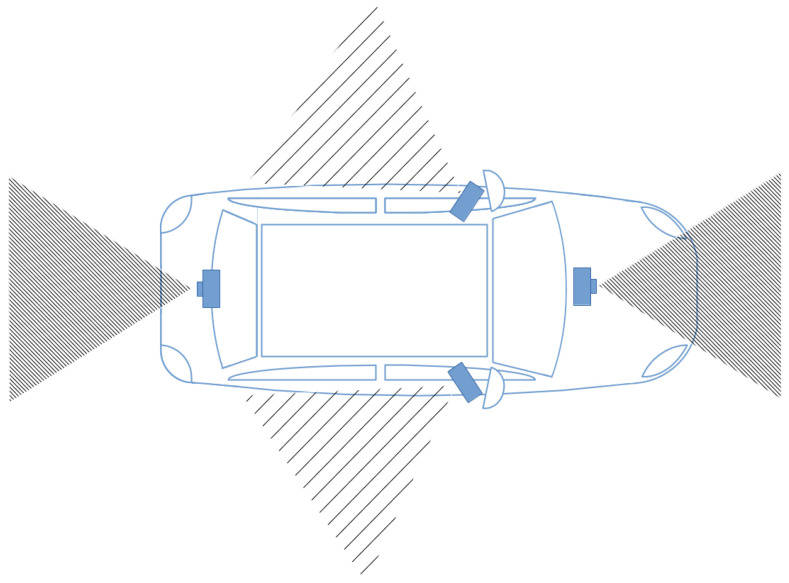
Illustration of the wireless sensors being placed in areas of interest for the ADAS.

Using an intra-vehicle wireless sensor network, structured by a Wi-Fi Direct and Bluetooth topology, a low-cost ADAS alternative extends a smartphone’s sensory perception by using a camera-based wireless sensor network. As previously shown in Figure 1, the Bluetooth sensors and Wi-Fi sensors communicate directly with the smartphone. A smartphone’s processing power is harnessed, where the sensors are placed around the vehicle at areas of interest shown, as in Figure 2. The figure contains vision sensors with a focus on the front and the rear of the vehicle. The sensors on either lateral side of the vehicle ensure that the vehicle’s blind spots are covered.

**Figure 3:**
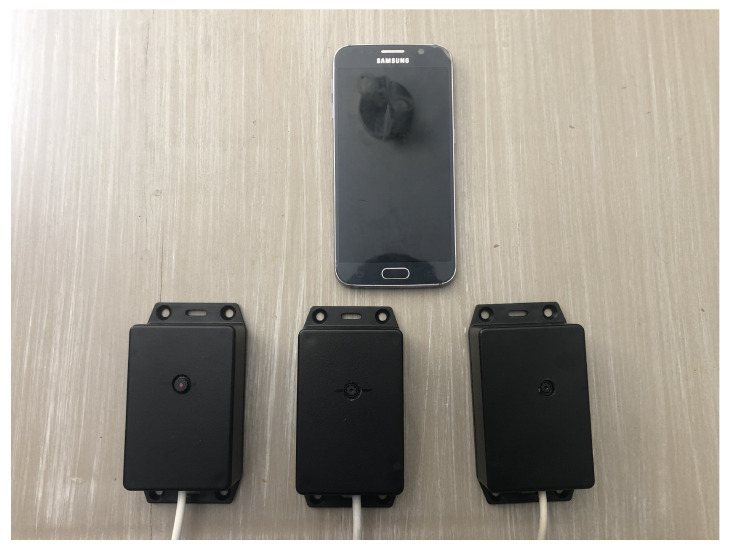
Android device and the three camera node sensors.

An IVWMSN network for an intra-vehicle environment that uses a low-cost camera, blind spot nodes, and a smartphone device, is discussed in the following section. By using smartphone-implemented Wi-Fi technologies, such as Wi-Fi Direct, the smartphone can act as the network host onto which video sensors can directly connect without adding a physical router as part of the architecture. By using the Wi-Fi protocol, higher transfer rates can send sensory data from a video sensor to enable advanced object detection around a vehicle. Three camera node prototypes and an Android phone are shown in Figure 3.

Video streaming from the network can then be used for real-time object detection applications for an ADAS on a smartphone device. Detection has successfully been carried out on video being sourced from a simulated video stream and network-sourced video streams. Due to large models not being able to run on smaller embedded devices, such as smartphones, they used a faster and lighter CNN, such as MobileNet, instead.

Lane detection and collision avoidance have been implemented for the ADAS, running on Android. It has been shown that a low-cost ADAS, using a smartphone, can carry out image processing techniques capable of detecting lanes on a real-time video stream. It has also been shown that a low-cost ADAS, using a smartphone, is able to carry out object detection techniques where distance estimation and collision risk can be calculated on a real-time video stream. The real-time video stream being sourced from the intra-vehicular wireless video network, is used to assist the driver by detecting lanes and warning the driver when he/she is moving closer to the centre of that lane. The implemented system helps the driver by warning him/her visually on the screen, i.e., about lane divergence to the left or right of the vehicle. The real-time video stream also assists the driver by warning the driver when his/her vehicle is moving closer to hazardous objects. The implemented system helps the driver by warning him/her visually on the screen about a possible collision in the front or rear of the vehicle.

### 2.1. Intra-Vehicular Wireless Multimedia Sensor Network

#### 2.1.1. Network Topology

**Figure 4:**
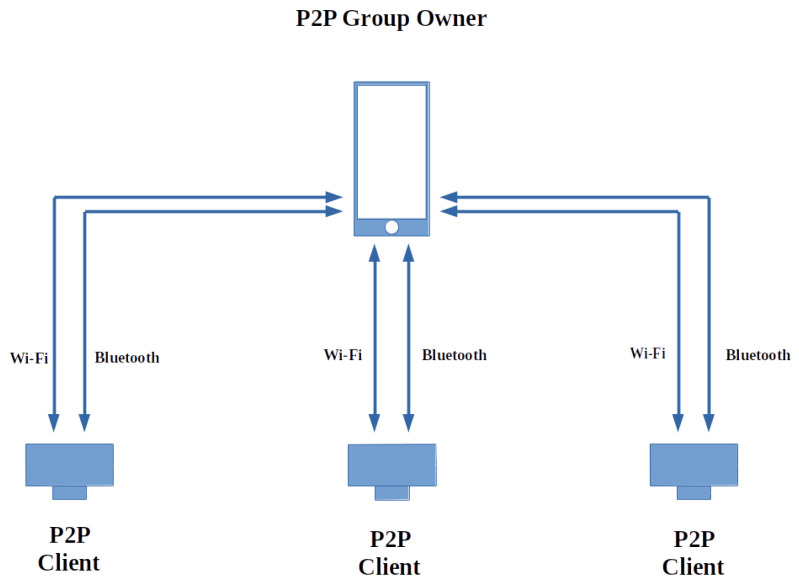
P2P topology 1:n, used by the camera nodes and smartphone.

The network’s main objective is to supply video feed to the processing unit, which is the Android device in the topology previously described. Wi-Fi Peer-to-Peer (P2P), also known as Wi-Fi Direct, is used in the design architecture to avoid having to use a physical router in the topology. A P2P 1:n topology is used where multiple clients are connected to one group owner with a single SSID, with a single security domain [47]. Not all devices support Wi-Fi Direct, but group owners support client legacy devices that fall outside the directional multi-gigabit (DMG) support. The transfer will still operate at IEEE 802.11 g or newer 2.4 GHz, supporting maximum physical bit rates of 54 Mbit/s. The legacy device supporting Wi-Fi identifies the group owner as a standard AP, as long as the smartphone supports Wi-Fi Direct. Most off-the-shelf Wi-Fi modules do not support Wi-Fi Direct, and using a legacy approach is more attainable. The basic topology is shown graphically in Figure 4.

**Figure 5:**
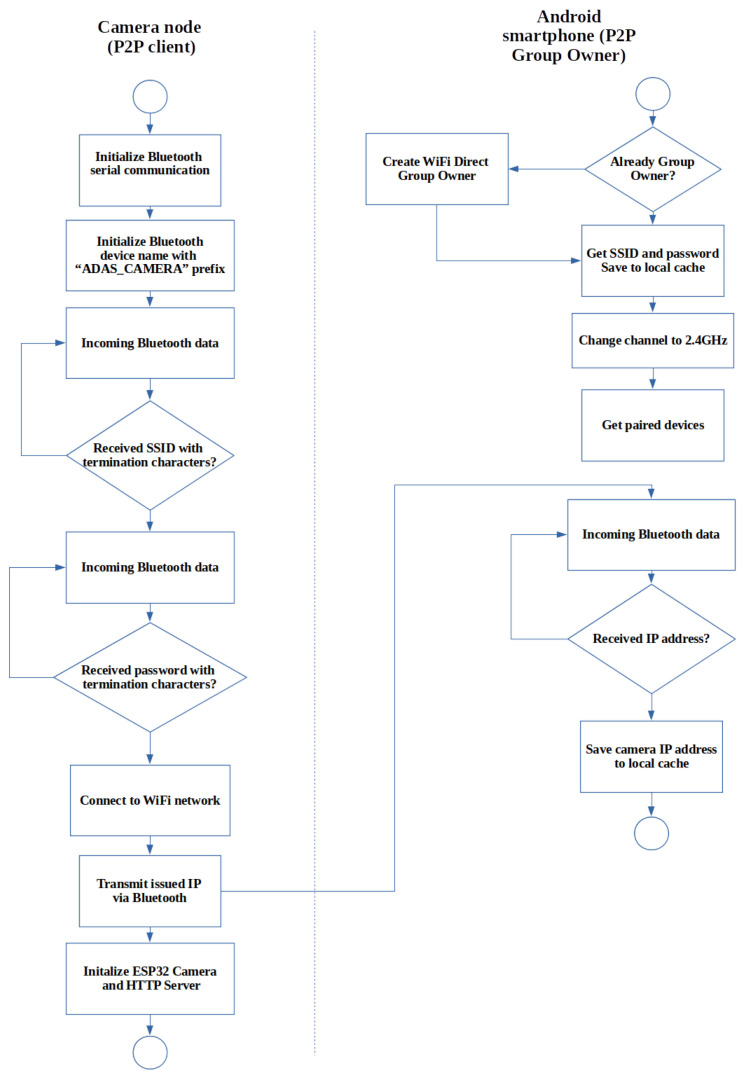
Network communication overview.

The network consists of another Bluetooth network used by both the blind spot sensors and vision sensors. The primary purpose of the Bluetooth network is discussed in the following sections. The camera nodes wait for the AP to start and then connect to the group owner. Depending on whether the network has been created before in the Android application’s life-cycle, the network can be re-established with a created SSID and passcode, but when the Android API creates a new network, a randomly generated SSID passcode is created. This caveat of Wi-Fi Direct prevents hard coding of the AP credentials to the camera node. A means of communication is needed to update the credentials on the camera nodes, in order to update network security details. Bluetooth pairing between the sensors and the smartphone is used for this communication. Bluetooth has shown unsatisfactory results when used for video transfer, but many low-cost Wi-Fi modules include Bluetooth communication as well [40]. Bluetooth protocol has been used as a serial communicator between devices, leaving the Wi-Fi protocol exclusively for video streaming. Using the Bluetooth serial communicator, information, such as the mentioned random generated passcode AP credentials, can be passed securely to the Bluetooth-paired camera nodes from the Android device. Figure 5 provides the overview of the communication between the camera node and Android smartphone.

#### 2.1.2. Bluetooth Communication

The Bluetooth protocol RFCOMM, in addition to L2CAP protocol, emulates an RS-232 serial port. The serial communication between the Android device and the camera node is used with a simple data stream and registered serial port service universally unique identifier (UUID) to allow communication and use of this service [48]. After the connection to the camera node through Bluetooth, protocol is established. The IP addresses are saved in the local cache of the Android application to be retrieved when streaming is initiated.

#### 2.1.3. Wi-Fi Communication

The network is managed and hosted by the Android application as the group owner. The camera nodes are seen as legacy devices as they do not support Wi-Fi Direct, but this does not negatively affect the architecture, since other intricate Wi-Fi Direct features are not needed. The camera node works within 2.4 GHz networks only. Such a 2.4 GHz network creation will cause miscommunication if the group owner establishes a network set at newer 5 GHz networks, as seen on high-end Android devices, which will cause the scan phase that has been initiated by the camera node to fail in connecting to the network. For this reason, the Android application is set to change the bandwidth on the creation of the network at 2.4 GHz channel 1 between the frequency range 2401–2423 MHz. Other channels, 1 to 14, can be explored if the need arises. The Wi-Fi network is hosted in a background thread of the Android application.

#### 2.1.4. Video Streaming

The camera nodes host an HTTP server that serves JPEG images from the CMOS camera. Once the camera node is connected to the network, the Android application can request data from the camera node, using the IP address acquired from the previously mentioned Bluetooth engagement. The images taken by the CMOS camera are streamed through TCP on the Wi-Fi network that is hosted by the group owner. A port and HTTP end-point are opened on the camera node HTTP server, allowing GET requests with multipart content-type “multipart/x-mixed-replace” to stream the image frames to the mobile phone. A boundary parameter is passed to the content-type as a delimiter to separate body parts of data, coming from frames [49]. The connection is kept open as long as the client requests packets, allowing the motion-JPEG (M-JPEG) stream to be processed by the Android device. The M-JPEG is not handled by the Android device as a video file, but is received as an image bitmap instead. A raw bitmap supports future object detection implementation without stripping frames from a video file, thereby requiring extra processing on the Android application.

**Figure 6:**
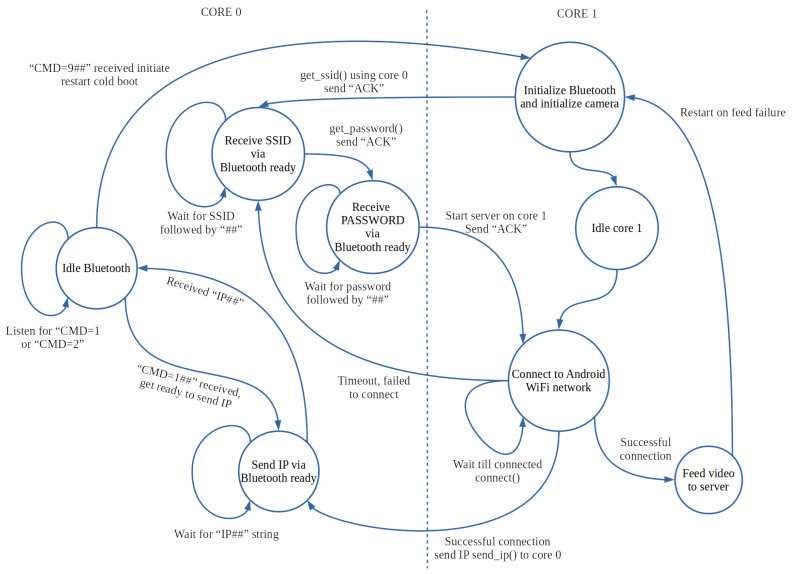
State machine of two cores for communication and dedicated video feed.

An ADAS requires real-time capabilities concerning where delays can be hazardous to the driver. The safety of the driver is an uncompromising factor that needs to take priority. Acceleration and deceleration time (ADRT) is used when the driver adjusts his/her speed. The time difference between the driver having received the visual signal, and his/her reaction, needs to be slower than the video feed, which alternatively would serve as no use to the driver [40]. For the video streams to be helpful in a real-time application, the frame rate experienced by the driver on the ADAS assistance display should be at a minimal delay. Previous testing calling tasks, such as Bluetooth communication, has caused the Android video output view experience to be staggered, due to shared processing. The camera capturing an HTTP server was placed on a dedicated core on the camera node’s microcontroller to prioritise the video stream, in order to improve upon this problem. Another interaction, such as Bluetooth communication, was placed on another core with lower prioritisation. Prioritisation and core tainting were conducted by using FreeRTOS, a real-time operating system kernel for embedded devices and the previously used microcontroller’s dual-core capability. Each camera node consists of the state machine, as shown in Figure 6.

#### 2.1.5. Camera Node

**Figure 7:**
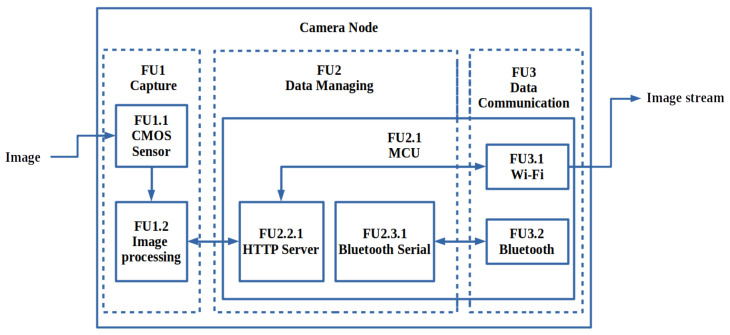
Functional diagram of the camera node, transferring the image stream over Wi-Fi.

The functional diagram shown in Figure 7 illustrates the design of the camera node. A low-cost OV2640 CMOS image sensor (FU1.1) captures images with an image array capable of maximum image transfer rates of 30 fps at SVGA and up to 60 fps in CIF [5]. The OV2640 camera chip also gives users control over image quality and formatting for future results optimisations (FU1.2). The off-the-shelf component ESP32-CAM development board that is used contains the aforementioned image sensor, as well as the ESP32 32 bit microcontroller (FU2.1), capable of running the HTTP server housed with the Wi-Fi MAC and Bluetooth controller (FU3.1 and FU3.2).

#### 2.1.6. Design Schematics and PCB Layouts

**Figure 8:**
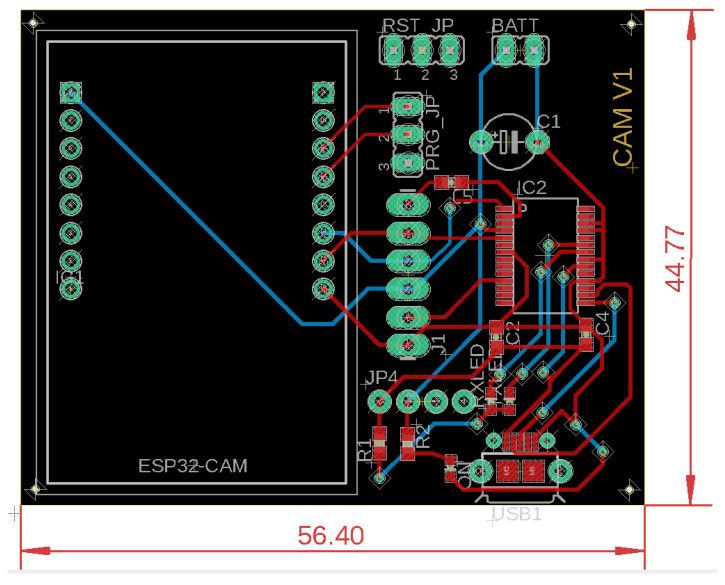
Designed camera node PCB board for ESP32-CAM breakout.

**Figure 9:**
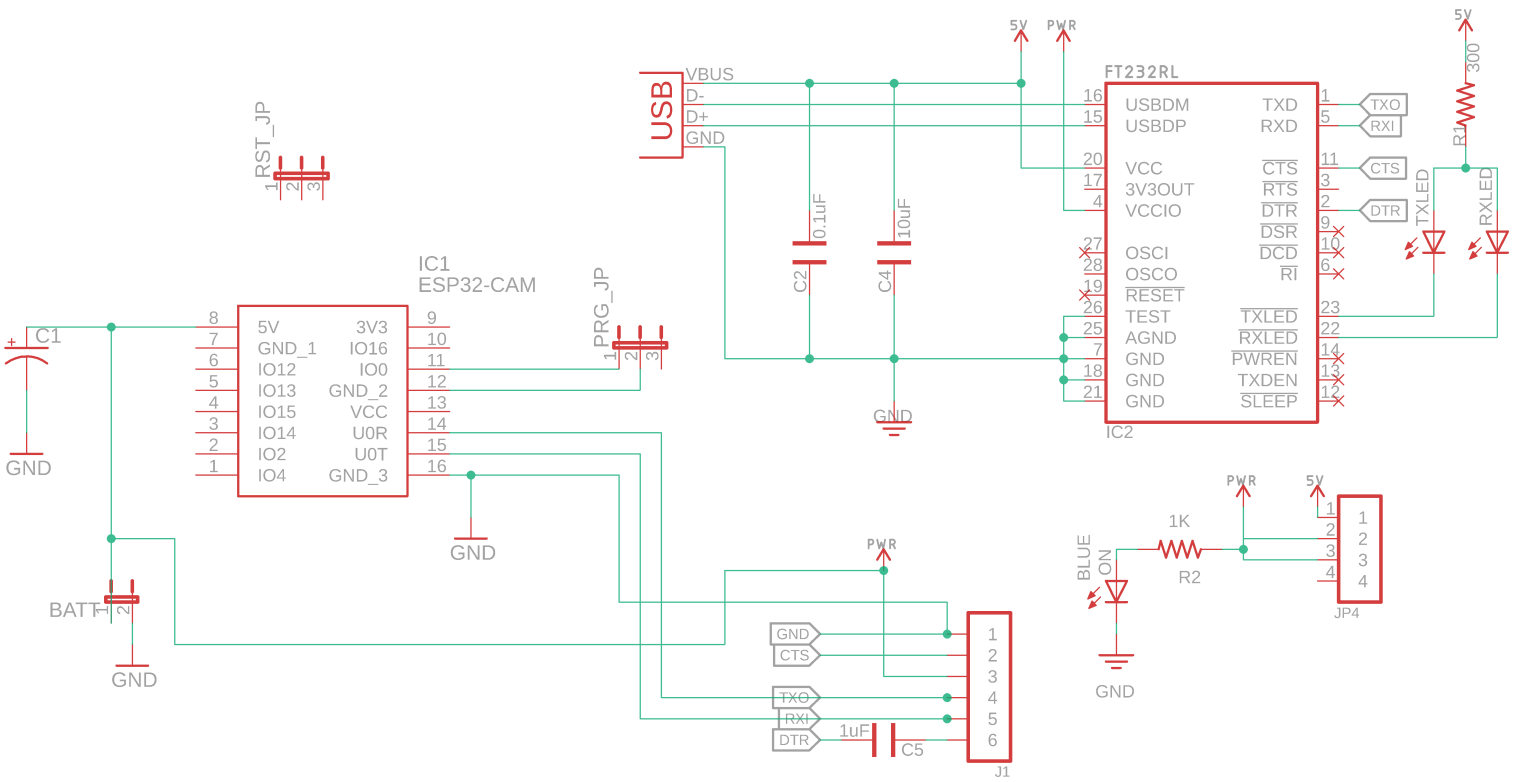
Schematic of the camera node.

A breakout board for the ESP32-CAM board has been designed that allows the development board to mount the camera node to a vehicle PCB, shown in Figure 8. The camera node should be able to withstand outdoor elements to be able to take proper field readings. The PCB is concealed in a protective container with ingress protection (IP), protected from dust and water. Figure 9 shows the designed schematic, where FT232RL IC is a TTL/RS232 converter used to program the ESP32 through a USB connection. The camera node consumes power through the USB connection, but jumpers have been placed on consuming battery power for future field testing.

#### 2.1.7. Camera Node Embedded Logic

The camera node’s software was written in C to communicate with the ESP32 microcontroller. Before the camera node hosts the HTTP server, the Bluetooth controller is engaged by starting the Bluetooth serial service by broadcasting the device name. The device name is prefixed with “ADAS_CAMERA”, which the Android application uses to filter paired devices for the ADAS application. The camera nodes remain in this state until the Android application sends the aforementioned SSID and passcode that has been terminated by consecutive 0x23 hex values to indicate the successful transfer for both the SSID and passcode.

After credentials are received successfully, the camera is initialised, and the Wi-Fi module attempts to connect to the Android-hosted network. If a successful connection to the network is complete, the local IP of the camera node is transmitted to the Android application, and the node’s HTTP server starts. The lightweight HTTP server creates a listening socket on TCP for HTTP traffic, where user-registered handlers are invoked, and sends back HTTP response packets to the Android application. The server has one purpose, which is to feed the image feed from the camera, where the server’s port socket is set at 80, and the user-registered handler is an HTTP GET method at the root “/”. The Android application then requests the feed from the URL “http://<camera node ip>:80”. When the user-registered handler is invoked, an image is requested from the camera and converted to JPEG compression, which is then sent to the Android application. The invoked handler continues this process indefinitely until the connection is closed by the smartphone client.

**Figure 10:**
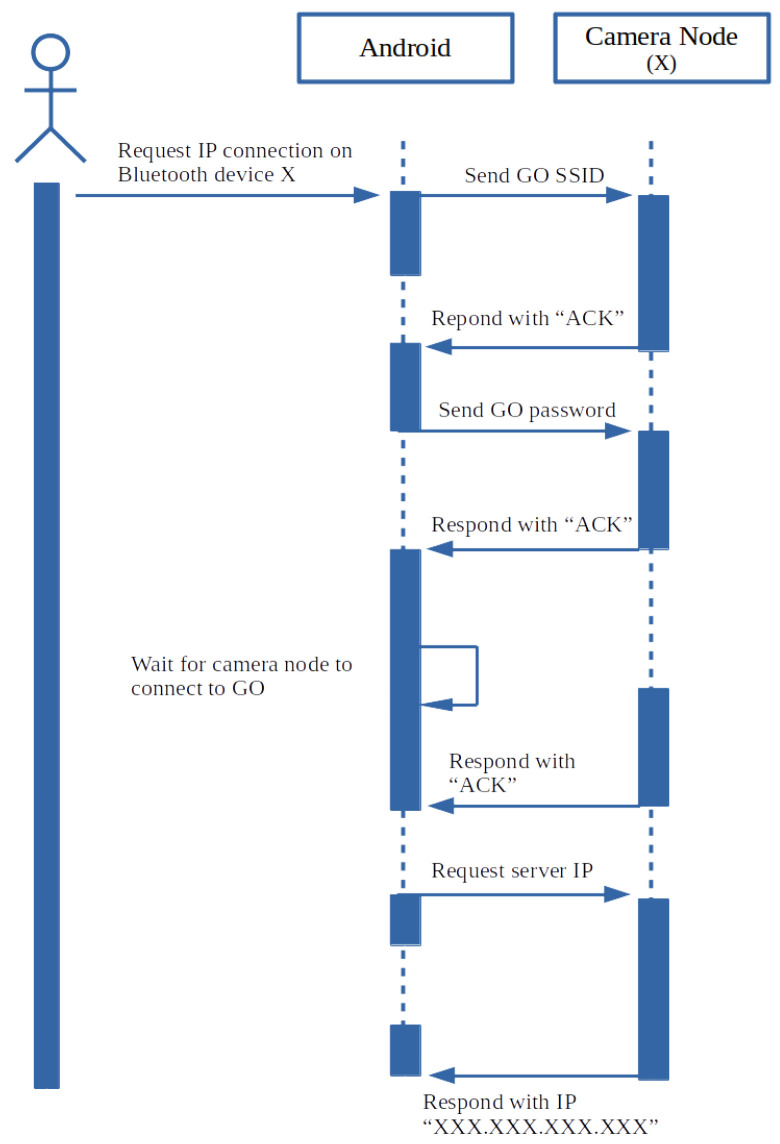
Bluetooth communication between the Android application and camera node.

Upon booting of the camera node, the Bluetooth communication is initialised with Wi-Fi communication. This only happens once during the camera node’s life cycle, but this can fall over to a restart if a failure occurs, such as brownouts or system locks. Added handshaking is implemented to force the video streaming to restart on the Android device command “CMD=9”. This mechanism of using “CMD” with a number is reserved to carry out other functionalities in the future. After initialisation, FreeRTOS is used to initiate a task pinned to core 0 of the microcontroller. This core deals with Bluetooth communication only. The state machine on core 0’s side receives the group owner’s (GO) SSID and password from the Android application by implementing the handshaking mechanism shown in Figure 10.

#### 2.1.8. Blind Spot Node

**Figure 11:**
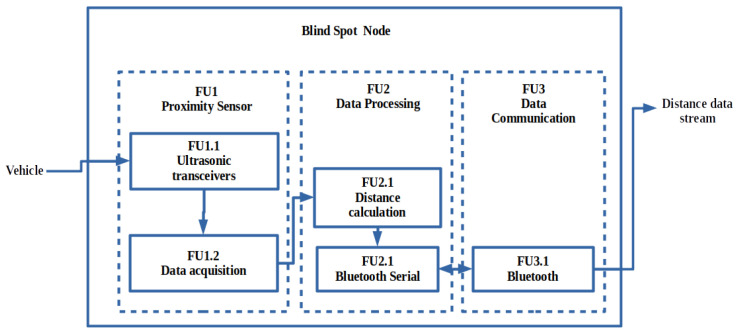
Functional diagram of the blind spot node, expressing the manner in which the distance measurement is streamed through Bluetooth.

The functional diagram shown in Figure 11 illustrates the design of the blind spot node. The node consists of three main components, namely the proximity sensor (FU1), microcontroller (FU2), and Bluetooth communication (FU3). Focusing on keeping the entire ADAS at a low cost, an ultrasonic sensor is used as the proximity sensor, which provides a 2 to 400 cm non-contact measurement with a ranging accuracy of 3 mm [50]. The ultrasonic sensor uses an echo and trigger mechanism that has been acquired by the microcontroller (MCU), where the distance is calculated by using the speed of sound. The same ESP32 development board, being used for the camera node from previous investigations, was used as the MCU of the blind spot node. The ESP32 32 bit microcontroller can run the Bluetooth controller (FU3.1), which is used to send through the calculated distance received from the ESP32 microcontroller and ultrasonic transceivers, using an RFCOMM Bluetooth serial.

**Figure 12:**
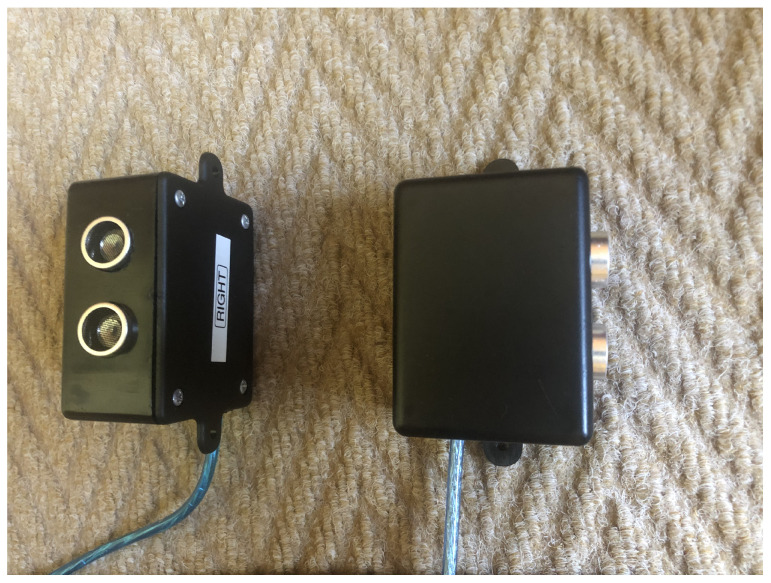
Left and right blind spot nodes.

The ultrasonic sensors pointing out from the side of the casing are directed to the blind spot area of the vehicle. The final prototype is shown in Figure 12.

### 2.2. Smartphone-Based ADAS

#### 2.2.1. Lane Detection

**Figure 13:**
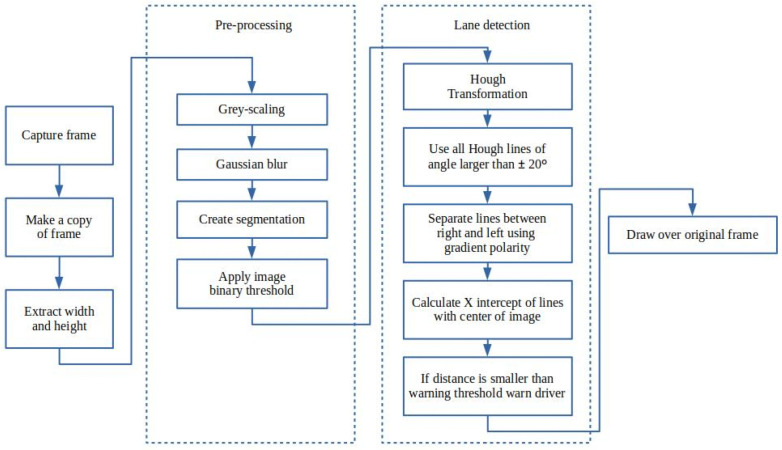
Functional diagram of the designed lane detection for lane assistance.

As an experimental implementation, the lane detection was written in Python, which was then written in JAVA on Android. OpenCV is a well-known library that focuses on real-time computer vision and was used in this implementation [51]. The same library is ported and available to the Android environment, where the same implementation logic is used for the Android development after a simulation experimentation is successful. The implemented Python streamer uses a previously developed simulator to simulate the oncoming road video stream. Each frame is captured and passed through the process shown by Figure 13.

An edge detection mechanism is used to detect lanes. Hough transform assists in detecting imperfect straight lines, which will provide further assistance when dotted lanes are at play.

In this implementation, the preprocessed frame is passed through a Hough transformation that returns an array of lines. A gradient is then calculated for each line in the array, where a slope smaller than a set acute angle is rejected. This slope is expressed by

(1) $$\theta =\left|\arctan(\frac{y_{2}-y_{1}}{x_{2}-x_{1}})\right|$$

By rejecting angles that are not within the set bounds, errors are avoided, such as horizontal lines that do not serve as possible road lanes.

Furthermore, to determine an estimate of where the vehicle is positioned relative to the lanes, a simple approach was taken by using the x-intercept of the detected lane and the centre of the width of the frame. The intercept of the lane was calculated by using the following

(2) $$x_{intercept}=\frac{x_{1}\nabla -y_{1}-I}{\nabla };\nabla =\frac{y_{2}-y_{1}}{x_{2}-x_{1}},$$

where *I* is the image height in pixels, assuming a Cartesian plane of the image where a (0,0) point starts at the top left corner, and the x-intercept is then used to calculate the distance from the centre by using half the width of the image. Essential lane assistance is then added, which will trigger a warning when the driver exceeds a certain tolerance of the left or right side lanes. The tolerance can be set as a percentage of half the width. Once the driver exits the safe zone, the driver will be warned that the vehicle is diverging lanes.

#### 2.2.2. Collision Detection

A multi-scale approach to optimise detection discovery by using different detection windows to detect objects in proximity to the driver’s vehicle was shown to deliver good results [9]. In this paper, however, “detection” does not use the windows as detection windows. Instead, it uses them as collision potentials because the detection was already done through a convolution neural network. The different regions are used as collision levels, namely near, intermediate, and far, to warn drivers that a collision could occur. The far region wraps the vanishing point area in the horizon, where the closer regions wrap a larger area of the images. The overlapping region is calculated for each detection to determine whether a detected box falls within a particular region. The two metrics $Overlap_{x}$ and $Overlap_{y}$ are used to compute the OverlapArea, which can be calculated as the product by

(3) $$Overlap_{x}=max\left(min\left(A_{x(right)};B_{x(right)}\right)-max\left(A_{x(left)};B_{x(left)}\right)\right)$$

(4) $$Overlap_{y}=max\left(min\left(A_{y(bottom)};B_{y(bottom)}\right)-max\left(A_{y(top)};B_{y(top)}\right)\right)$$

(5) $$OverlapArea=Overlap_{x}\times Overlap_{y}.$$

#### 2.2.3. Distance Estimation

**Figure 14:**
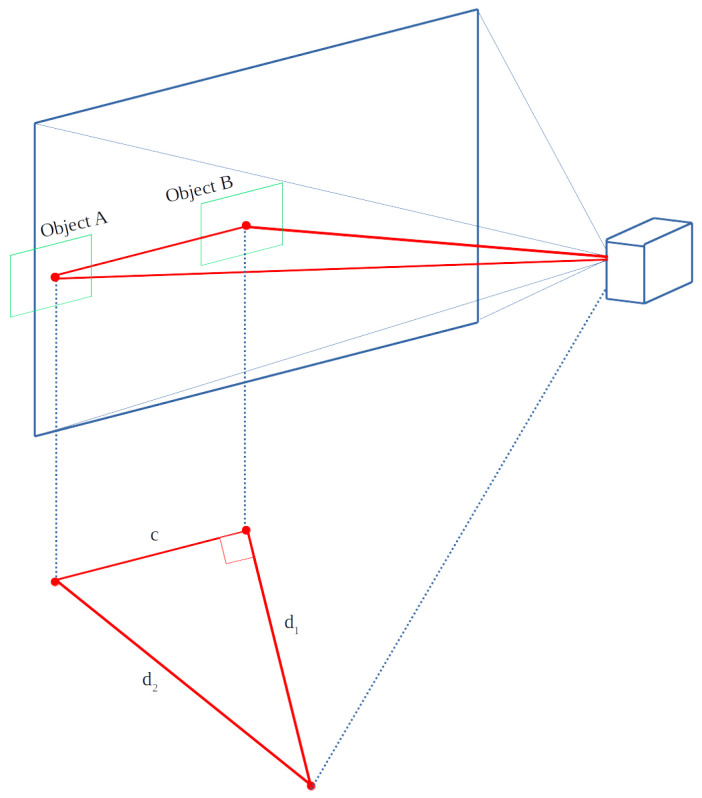
Projection of the camera, capturing the incoming scene.

A manual calibration process is required to determine the focal length, which is required before distance estimation can be carried out. After the focal length is calculated through a calibration process, the distance of the objects can be estimated by using the same equation, with the extension of adding a right angle calculation to determine the deviation from the centre of the image, which is better illustrated by Figure 14.

The figure shows how the distance is recalculated when an object has been detected and deviates from the centroid of the image. The recalculated object is calculated by using the adjusted equation

(6) $$d_{2}=\sqrt{{\left(\frac{width_{object}\times f}{width_{pixels}}\right)}^{2}+{\left(\left|CentroidX_{image}-CentroidX_{object}\right|\right)}^{2}}.$$

The distance to the centre of a detect box is calculated by using the aforementioned pinhole method. If the detected box deviates from the centre, the distance is adjusted by using Equation (6).

## 3. Results

### 3.1. Simulated Environment

Tests were carried out, using different environments. Firstly, the nodes were simulated by using simulator nodes, hosting streams at different resolutions. The different streams consisted of low, medium, high and ultra-high resolutions, tested at concurrent connection combinations of one to six streams. The simulator ensures that each stream acts as a camera node by looping the same video at different resolutions. Network performance was then shown for the actual hardware camera nodes in a controlled environment to illustrate the manner in which the network and camera nodes performed without an intra-vehicle environment. Lastly, an intra-vehicle environment was used where camera nodes were placed inside the vehicle’s front and rear to take the same readings as the controlled environment.

Six streams were run, from one to six concurrent streams at different resolutions, and then the averages of the frame rates per second were calculated, as shown in Table 1. At the lowest resolution, frame rates were the highest, with decreasing frame rates as the streams increased. As the resolutions increased, frame rates dropped to rates that did not accommodate real-time applications.

At high resolutions, the current solution cannot support an ADAS. Improved transmission methods, such as HTTP live streaming (HLS) and real time streaming protocol (RTSP), would improve this by incorporating improved encoding and decoding strategies, but would require more demanding hardware and increased costs.

### 3.2. Intra-Vehicle Environment

**Figure 15:**
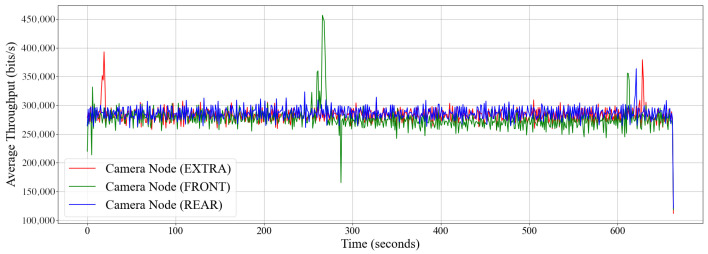
Throughput of three camera nodes in an intra-vehicle environment where readings were taken over 10 min.

**Figure 16:**
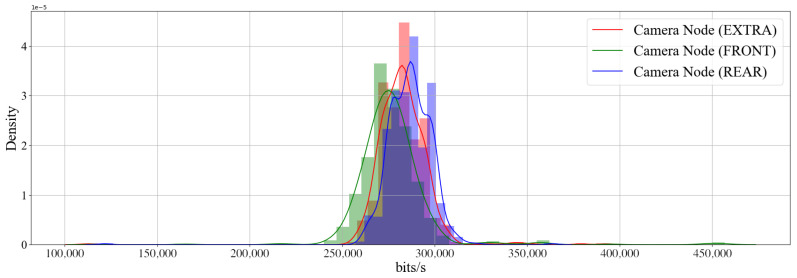
Density plot of the throughput of three camera nodes in an intra-vehicle environment over 10 min.

**Figure 17:**
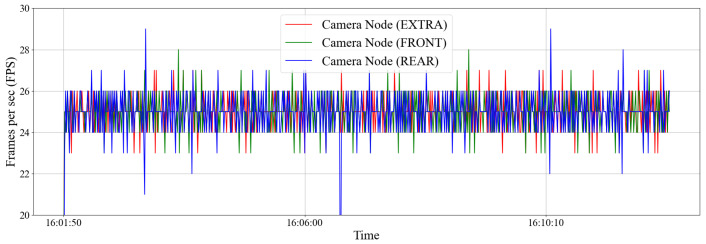
Frame rate of all three camera nodes mounted on the vehicle, being fed to the Android device over a 10-minute period.

The intra-vehicle environment was tested by placing the sensors externally at the front and at the rear of the vehicle. The tests were carried out on a stationary vehicle. An extra camera node was also placed on the passenger seat to be compared to the controlled environment. Readings were then taken during the 10 min. The smartphone was mounted inside the vehicle at the driver’s seat near the vehicle’s dashboard. Figure 15 shows the throughput of all three sensors and Figure 16 reveals the density plot. The throughput of the network hovers at under 300 kbit/s. Figure 17 shows the frame rate of all three camera nodes fed to the Android device.

### 3.3. Android Battery Consumption

**Figure 18:**
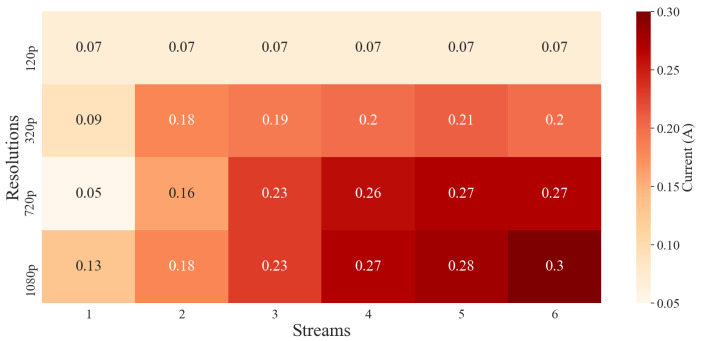
A heat map of average current draws on the Android device while running a number of streams.

Multiple streams, as well as different resolutions, were tested to record current draws at different combinations. Figure 18 shows the matrix of current draw averages at different stream to resolution combinations. As expected, as streams and resolutions increased, the current draw increased, due to more processing on the smartphone.

### 3.4. Testing Environment

#### 3.4.1. Collision Avoidance

**Figure 19:**
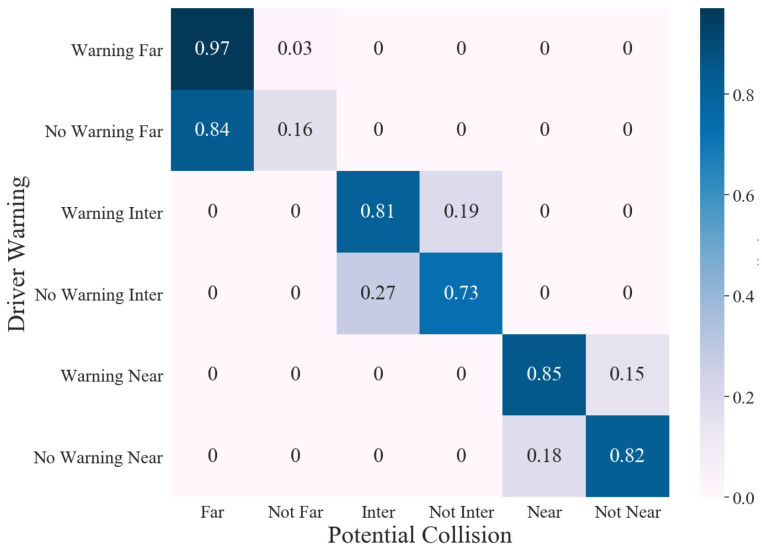
Confusion matrix of three detection regions and their binary outcomes.

Collision detection uses a MobileSSDv1 model, pre-trained on the COCO dataset to detect vehicles from the video network stream. The detector uses three different areas to inform the driver about a potential collision. The performance of the detector was tested by implementing the Bosch Boxy data set, which consists of annotated vehicles for training and evaluating object detection methods for self-driving cars on freeways [52]. By comparing the annotated frames with the detection carried out by the collision detector, a confusion matrix, as shown in Figure 19, was constructed. The matrix was divided into three regions to illustrate different binary outcomes.

#### 3.4.2. Distance Detection

A test vehicle was used to carry out the distance estimation algorithm. Three different intervals were marked on the road at 3 metre intervals. The focal length was calibrated at a measured 9 metres, and then the distance was estimated at ground truth measurements of 3, 6, and 9 metres, respectively. Table 2 shows the different intervals, and the accuracies of the readings after the distance estimation was carried out. Distance errors aggravate as the distance from the original 9 metre calibration point changes.

#### 3.4.3. Lane Detection

**Figure 20:**
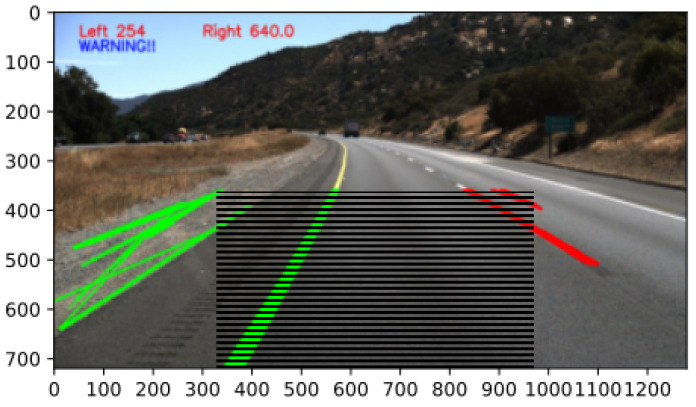
An example of a left lane warning where the hatched area represents the tolerance of the position where lanes can be detected.

Forming part of the ADAS, the driver is warned when potentially entering a lane on the left or right. If the warning tolerance is too high, then every lane being detected will fire a warning to the driver. Too high a tolerance is not ideal because only lanes close to the vehicle, which are assumed as drifting of the vehicle, should be used to warn the driver. On the other hand, if the tolerance is too low, the detected lanes will never enter the tolerance area that should trigger a warning. The tolerance is set at an estimate of the width of the ADAS-equipped vehicle. Figure 20 shows the area where warnings to the driver will be fired if the lane’s x-intercepts fall within the hatched areas.

Accuracy is the number of correctly classified lanes, divided by the total number of examples in the test set. Accuracy is helpful but does not cater for subtleties of imbalances or weighting of false negatives and false positives. The F-score helps to solve this by placing more emphasis on recall or precision. By setting β=2, recall is twice as important as precision, considering that it is much worse to miss a lane than to give a false alert for a non-existing lane.

(7) $$F_{score}=\frac{(1+\beta^{2})TP}{\left(1+\beta^{2}\right)TP+\beta^{2}FN+FP}=0.94$$

(8) $$Accuracy=\frac{TP+TN}{samples}=96\%$$

#### 3.4.4. Blind Spot Detection

**Figure 21:**
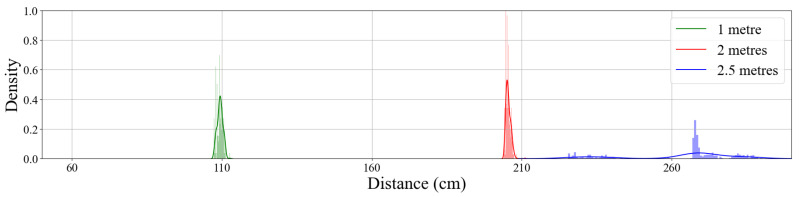
Distribution plot for samples measured at two different distance intervals to the car.

A distance experiment was set up where intervals separated by a metre, starting from the vehicle, were tested, to test the accuracy and performance of the developed blind spot nodes. Raw Bluetooth distance sample outputs were taken at every metre interval, using the blind spot node and a Bluetooth Android terminal application. Figure 21 shows the distribution plots of two intervals at 1, 2, and 2.5 metres, respectively. The samples decrease at distances larger than 2.5 metres, due to the ultrasonic sensors diminishing echo signal returns. The accuracy also decreases as the distance increases. The distance accuracy degradation is expected, as the sensor’s specifications only support 2 metres.

### 3.5. Android Application

By using the Wi-Fi protocol, higher transfer rates can send sensory data from a video sensor to enable advanced object detection around a vehicle. Video streaming from the network can then be used for real-time object detection applications for an ADAS on a smartphone device. Detection was successfully carried out on video being sourced from a simulated video stream and network-sourced video streams. Due to large models not being able to run on smaller embedded devices, such as smartphones, they used a faster and lighter CNN, such as MobileNet, instead.

**Figure 22:**
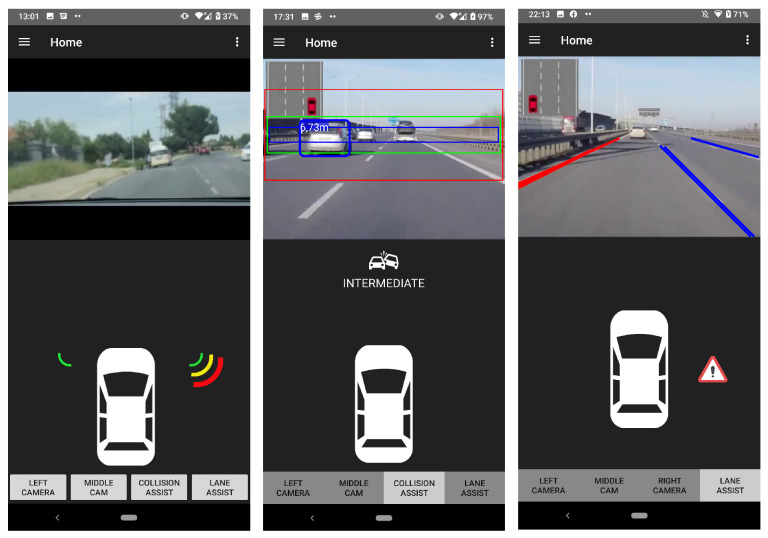
Screenshots of different detection types running the ADAS on Android.

Lane detection and collision avoidance were implemented for the ADAS, running on Android. It has been shown that a low-cost ADAS, using a smartphone, can carry out image processing techniques capable of detecting lanes on a real-time video stream. It has also been shown that a low-cost ADAS, using a smartphone, is able to carry out object detection techniques where distance estimation and collision risk can be calculated on a real-time video stream. The real-time video stream, being sourced from the intra-vehicular wireless video network, is used to assist the driver by detecting lanes and warning the driver when he/she is moving closer to the centre of that lane. The implemented system helps the driver by warning him/her visually on the screen about lane divergence to the left or right of the vehicle which can be seen in Figure 22. The real-time video stream also assists the driver by warning the driver when his/her vehicle is moving closer to hazardous objects. The implemented system helps the driver by warning him/her visually on the screen about a possible collision in the front or rear of the vehicle.

Blind spot detection is added to the implemented ADAS running on Android. It has been shown that a low-cost ADAS, using a smartphone, can carry out blind spot detection where ultrasonic sensors can detect the distance of a vehicle in the driver’s blind spot, in order to prevent a collision. The Bluetooth devices form an addition to the already developed network that provides a real-time video stream being sourced from the intra-vehicular wireless video network. The blind spot addition will assist the driver with the help of visual warnings on a screen if a possible collision in the blind spot of the vehicle is going to occur which can be seen in Figure 22.

**Figure 23:**
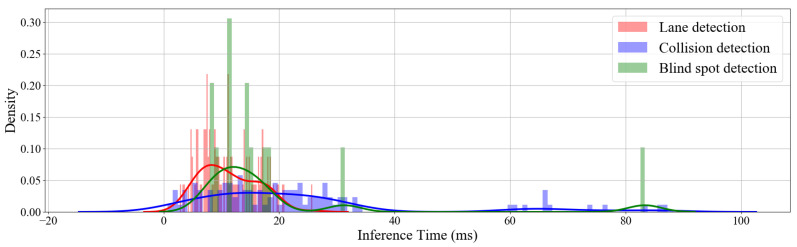
Density distribution plot, showing the processing times for collision, lane, and blind spot detection.

The collision and lane detection processing speeds are measured for each frame before and after detection. The sample rate of the blind spot detector is calculated by the number of packets received per second. Figure 23 shows the density plot of the lane, collision, and blind spot inference times for the ADAS, being run on a middle-range Android smartphone.

Collision detection has the most significant average delay processed on a middle-range Android smartphone, running the collision avoidance module. The network delay and the collision detection delay have caused the drop in frames but are still usable in a real-time application. A summary of the proposed system’s processing times is shown in Table 3, which includes the corresponding frame rates. The network delay is the most significant contributing factor to frames being dropped.

## 4. Conclusions

ADAS are prevalent in high-end vehicles, and low-cost ADAS have not been widely available. Most drivers have smartphones that are capable of carrying out complex processing, and can perform network communications. Smartphones are helpful in ADAS, but sensors are limited to the peripherals on the smartphone device. In this paper, a low-cost smartphone-based ADAS was developed using a high speed IVWMSN as the communication backbone, thereby extending the smartphone’s onboard sensors and using the smartphone as a processing platform. It was demonstrated how the proposed system consumes sensory data received from the IVWSN and performs accurate lane detection, collision detection, and blind spot detection in real-time, while Bluetooth communication was used for lower data rate sensors, such as proximity sensors. Data transmission rates required for camera-based sensors in WMSN were achieved. Data transmission rates and the performance of each individual ADAS function were analysed and found to be adequate for the enablement of a functional low-cost smartphone-based ADAS.

It was found that an inexpensive advanced driver-assistance system alternative can be conceptualised by using object detection techniques processed on a smartphone from multiple streams, sourced from an intra-vehicle wireless sensor network, composed of camera sensors. To allow the smartphone application to be used by a driver in real-time, the frame rate is required to be high enough to accommodate the user to react to an ADAS warning. Table 3 shows a summary of the average processing times of the proposed system, which uses the IVWSN network. The Wi-Fi network can reach high throughput rates, but real-time processing is a trade-off that can be improved by using encoding and compression techniques for transferring video streams at higher resolutions. Even though the detectors prefer lower resolution images, other WSN applications might benefit from transferring video streams at higher resolutions. More efficient management of video streams can be implemented to alleviate processing strain and power usage by the smartphone. A switching algorithm can accommodate multiple camera streams by focusing on detection areas of higher priority, depending on driving scenarios determined by the object detection.

## Figures and Tables

**Table 1:** Average frames per second of different resolutions at different streamed nodes.

Resolution	1 Stream	2 Streams	3 Streams	4 Streams	5 Streams	6 Streams
120p	522.22	265.91	175.64	131.91	107.31	87.73
320p	47.02	38.90	22.76	20.35	14.64	13.47
720p	3.57	2.65	2.43	2.02	1.71	1.52
1080p	1.04	1.00	1.00	1.00	1.00	1.00

**Table 2:** Results for a test vehicle with a width of 155 cm.

Test Interval	Actual Distance (cm)	Calculated Distance (cm)	Error (cm)	Accuracy %
3 metre mark	300	362	62	83%
6 metre mark	600	604	4	99%
9 metre mark	900	902	2	100%

**Table 3:** Processing times for the proposed system.

Delay Type	FPS	Average (ms)	Standard Deviation (ms)
Collision detection	44.1	22.7	20.1
Lane detection	82.6	12.1	4.8
Blind spot detection	65.7	15.2	25.4
Camera node (including network)	25.6	39	1.1

## Data Availability

Publicly available datasets were analysed in this study. There data can be found here: https://github.com/TuSimple/tusimple-benchmark and https://boxy-dataset.com/boxy/ accessed on 25 November 2021.

## Abbreviations

The following abbreviations are used in this manuscript:

ACC	autonomous cruise control
ADAS	advanced driver-assistance system(s)
ADRT	acceleration and deceleration time
BLE	Bluetooth Low Energy
CAN	controller area network(s)
CMOS	complementary metal-oxide semiconductor
CNN	convolutional neural network(s)
ECU	electronic control unit
FCA	forward collision avoidance
IVWSN	intra-vehicular wireless sensor network
LBP	local binary patterns
MOST	media-oriented system transport
SBZA	side blind zone alert
UWB	ultra-wideband
UUID	universally unique identifier
WMSN	wireless multimedia sensor network
YOLO	you only look once

## References

[1] Liu F., Sparbert J., Stiller C. IMMPDA vehicle tracking system using asynchronous sensor fusion of radar and vision. Proceedings of the 2008 IEEE Intelligent Vehicles Symposium.

[2] Wei P., Cagle L., Reza T., Ball J., Gafford J. (2018). LiDAR and camera detection fusion in a real-time industrial multi-sensor collision avoidance system. Electronics.

[3] Sole A., Mano O., Stein G.P., Kumon H., Tamatsu Y., Shashua A. Solid or not solid: Vision for radar target validation. Proceedings of the IEEE Intelligent Vehicles Symposium.

[4] Coue C., Fraichard T., Bessiere P., Mazer E. Multi-sensor data fusion using Bayesian programming: An automotive application. Proceedings of the Intelligent Vehicle Symposium.

[5] Bhoraskar R., Vankadhara N., Raman B., Kulkarni P. Wolverine: Traffic and road condition estimation using smartphone sensors. Proceedings of the 2012 Fourth International Conference on Communication Systems and Networks (COMSNETS 2012).

[6] Fazeen M., Gozick B., Dantu R., Bhukhiya M. (2012). Safe driving using mobile phones. IEEE Trans. Intell. Transp. Syst..

[7] Chaovalit P., Saiprasert C., Pholprasit T. A method for driving event detection using SAX on smartphone sensors. Proceedings of the 2013 13th International Conference on ITS Telecommunications (ITST).

[8] Mohan P., Padmanabhan V.N., Ramjee R. Nericell: Using mobile smartphones for rich monitoring of road and traffic conditions. Proceedings of the 6th ACM Conference on Embedded Network Sensor Systems.

[9] Romera E., Bergasa L.M., Arroyo R. A Real-Time Multi-scale Vehicle Detection and Tracking Approach for Smartphones. Proceedings of the 2015 IEEE 18th International Conference on Intelligent Transportation Systems.

[10] You C.W., Lane N.D., Chen F., Wang R., Chen Z., Bao T.J., Montes-de Oca M., Cheng Y., Lin M., Torresani L. Carsafe app: Alerting drowsy and distracted drivers using dual cameras on smartphones. Proceedings of the 11th Annual International Conference on Mobile Systems, Applications, and Services.

[11] Petrovai A., Danescu R., Nedevschi S. A stereovision based approach for detecting and tracking lane and forward obstacles on mobile devices. Proceedings of the 2015 IEEE Intelligent Vehicles Symposium (IV).

[12] Viola P., Jones M. Rapid object detection using a boosted cascade of simple features. Proceedings of the 2001 IEEE Computer Society Conference on Computer Vision and Pattern Recognition.

[13] Bhandari R., Nambi A.U., Padmanabhan V.N., Raman B. DeepLane: Camera-assisted GPS for driving lane detection. Proceedings of the 5th Conference on Systems for Built Environments.

[14] Girshick R., Donahue J., Darrell T., Malik J. Rich feature hierarchies for accurate object detection and semantic segmentation. Proceedings of the IEEE Conference on Computer Vision and Pattern Recognition.

[15] Girshick R. Fast R-CNN. Proceedings of the IEEE International Conference on Computer Vision.

[16] Ren S., He K., Girshick R., Sun J. (2017). Faster R-CNN: Towards Real-Time Object Detection with Region Proposal Networks. IEEE Trans. Pattern Anal. Mach. Intell..

[17] Redmon J., Divvala S., Girshick R., Farhadi A. You only look once: Unified, real-time object detection. Proceedings of the IEEE Conference on Computer Vision and Pattern Recognition.

[18] Howard A.G., Zhu M., Chen B., Kalenichenko D., Wang W., Weyand T., Andreetto M., Adam H. (2017). Mobilenets: Efficient convolutional neural networks for mobile vision applications. arXiv.

[19] Aly H., Basalamah A., Youssef M. Lanequest: An accurate and energy-efficient lane detection system. Proceedings of the 2015 IEEE International Conference on Pervasive Computing and Communications (PerCom).

[20] Lan M., Rofouei M., Soatto S., Sarrafzadeh M. SmartLDWS: A robust and scalable lane departure warning system for the smartphones. Proceedings of the 2009 12th International IEEE Conference on Intelligent Transportation Systems.

[21] Tang S.J.W., Ng K.Y., Khoo B.H., Parkkinen J. Real-time lane detection and rear-end collision warning system on a mobile computing platform. Proceedings of the 2015 IEEE 39th Annual Computer Software and Applications Conference.

[22] Bergasa L.M., Almería D., Almazán J., Yebes J.J., Arroyo R. Drivesafe: An app for alerting inattentive drivers and scoring driving behaviors. Proceedings of the 2014 IEEE Intelligent Vehicles Symposium Proceedings.

[23] Lin J.R., Talty T., Tonguz O.K. (2015). A blind zone alert system based on intra-vehicular wireless sensor networks. IEEE Trans. Ind. Informatics.

[24] Wu B.F., Huang H.Y., Chen C.J., Chen Y.H., Chang C.W., Chen Y.L. (2013). A vision-based blind spot warning system for daytime and nighttime driver assistance. Comput. Electr. Eng..

[25] Lin B.F., Chan Y.M., Fu L.C., Hsiao P.Y., Chuang L.A., Huang S.S., Lo M.F. (2012). Integrating appearance and edge features for sedan vehicle detection in the blind-spot area. IEEE Trans. Intell. Transp. Syst..

[26] Chen C., Chen Y. Real-time approaching vehicle detection in blind-spot area. Proceedings of the 2009 12th International IEEE Conference on Intelligent Transportation Systems.

[27] Singh S., Meng R., Nelakuditi S., Tong Y., Wang S. SideEye: Mobile assistant for blind spot monitoring. Proceedings of the 2014 International Conference on Computing, Networking and Communications (ICNC).

[28] López A.M., Imiya A., Pajdla T., Álvarez J.M. (2017). Computer vision in vehicle technology: Land, sea, and air. Computer Vision in Vehicle Technology: Land, Sea, and Air.

[29] Dong Y., Hu Z., Uchimura K., Murayama N. (2010). Driver inattention monitoring system for intelligent vehicles: A review. IEEE Trans. Intell. Transp. Syst..

[30] Allamehzadeh A., de la Parra J.U., Hussein A., Garcia F., Olaverri-Monreal C. Cost-efficient driver state and road conditions monitoring system for conditional automation. Proceedings of the 2017 IEEE Intelligent Vehicles Symposium (IV).

[31] Qiao Y., Zeng K., Xu L., Yin X. A smartphone-based driver fatigue detection using fusion of multiple real-time facial features. Proceedings of the 2016 13th IEEE Annual Consumer Communications & Networking Conference (CCNC).

[32] He J., Roberson S., Fields B., Peng J., Cielocha S., Coltea J. (2013). Fatigue detection using smartphones. J. Ergon..

[33] Lee B.G., Chung W.Y. (2012). A smartphone-based driver safety monitoring system using data fusion. Sensors.

[34] Smirnov A., Kashevnik A., Lashkov I., Baraniuc O., Parfenov V. Smartphone-based identification of dangerous driving situations: Algorithms and implementation. Proceedings of the 2016 18th Conference of Open Innovations Association and Seminar on Information Security and Protection of Information Technology (FRUCT-ISPIT).

[35] Tuohy S., Glavin M., Hughes C., Jones E., Trivedi M., Kilmartin L. (2014). Intra-vehicle networks: A review. IEEE Trans. Intell. Transp. Syst..

[36] Green R.J., Rihawi Z., Mutalip Z.A., Leeson M.S., Higgins M.D. Networks in automotive systems: The potential for optical wireless integration. Proceedings of the 2012 14th International Conference on Transparent Optical Networks (ICTON).

[37] Iturri P.L., Aguirre E., Azpilicueta L., Garate U., Falcone F. (2014). ZigBee radio channel analysis in a complex vehicular environment [wireless corner]. IEEE Antennas Propag. Mag..

[38] Rahman M.A. (2014). Design of wireless sensor network for intra-vehicular communications. International Conference on Wired/Wireless Internet Communications.

[39] Rahman M.A., Ali J., Kabir M.N., Azad S. (2017). A performance investigation on IoT enabled intra-vehicular wireless sensor networks. Int. J. Automot. Mech. Eng..

[40] Lee J.S., Su Y.W., Shen C.C. A comparative study of wireless protocols: Bluetooth, UWB, ZigBee, and Wi-Fi. Proceedings of the IECON 2007-33rd Annual Conference of the IEEE Industrial Electronics Society.

[41] Akyildiz I.F., Melodia T., Chowdhury K.R. (2007). A survey on wireless multimedia sensor networks. Comput. Netw..

[42] Kato T., Ninomiya Y., Masaki I. (2002). An obstacle detection method by fusion of radar and motion stereo. IEEE Trans. Intell. Transp. Syst..

[43] Zhang F., Clarke D., Knoll A. Vehicle detection based on LiDAR and camera fusion. Proceedings of the 17th International IEEE Conference on Intelligent Transportation Systems (ITSC).

[44] Khan J. Using ADAS sensors in implementation of novel automotive features for increased safety and guidance. Proceedings of the 2016 3rd International Conference on Signal Processing and Integrated Networks (SPIN).

[45] Mahapatra R., Kumar K.V., Khurana G., Mahajan R. Ultra sonic sensor based blind spot accident prevention system. Proceedings of the 2008 International Conference on Advanced Computer Theory and Engineering.

[46] Akhlaq M., Sheltami T.R., Helgeson B., Shakshuki E.M. (2012). Designing an integrated driver assistance system using image sensors. J. Intell. Manuf..

[47] (2016). Wi-Fi Alliance. Wi-Fi Peer-to-Peer (P2P) Technical Specification Version 1.7. https://documents.pub/document/wi-fi-p2p-technical-specification-technical-specification-version-17-this-document.html.

[48] “Bluetooth Overview-Key Classes and Interfaces”. Android Developers Documentation Guides. https://developer.android.com/guide/topics/connectivity/bluetooth.

[49] “HTTP-Hypertext Transfer Protocol”. W3C Architecture Domain. https://www.w3.org/Protocols/.

[50] (2013). Ultrasonic Ranging Module HC-SR04. https://cdn.sparkfun.com/datasheets/Sensors/Proximity/HCSR04.pdf.

[51] “OpenCV Releases”. OpenCV.org. https://opencv.org/releases.

[52] Behrendt K. Boxy Vehicle Detection in Large Images. Proceedings of the IEEE International Conference on Computer Vision Workshops.

